# A minute primary gastric synovial sarcoma with ulcer: a case report

**DOI:** 10.1186/s13000-021-01175-3

**Published:** 2021-12-13

**Authors:** Kanako Yoshiyasu, Hiroshi Kono, Yudai Hojo, Yoshinori Ishida, Akio Tamura, Keisuke Nakai, Tadayuki Oshima, Hiroto Miwa, Hisashi Shinohara, Seiichi Hirota

**Affiliations:** 1 Department of Surgical Pathology, Hyogo College of Medicine, 1-1 Mukogawacho, 663-8501 Nishinomiya, Hyogo Japan; 2 Upper Gastrointestinal Division, Department of Surgery, Hyogo College of Medicine, 1-1 Mukogawacho, 663-8501 Nishinomiya, Hyogo Japan; 3 Division of Gastroenterology and Hepatology, Department of Internal Medicine, Hyogo College of Medicine, 1-1 Mukogawacho, 663-8501 Nishinomiya, Hyogo Japan

**Keywords:** Synovial sarcoma, Monophasic fibrous type, Stomach, Fusion gene analysis, SS18-SSX1

## Abstract

**Background:**

Synovial sarcomas are a rare type of high-grade sarcomas with unknown cell origin. They arise predominantly in the soft tissues but rarely in the stomach. We recently encountered a rare case of minute gastric synovial sarcoma.

**Case presentation:**

A 61-year-old Japanese woman was pointed out edematous erosion at the body of the stomach. Biopsy specimen showed dense proliferation of spindle-shaped tumor cells mixed with smooth muscle fibers of the muscularis mucosae. Although the definite histological diagnosis was undetermined, the patient underwent laparoscopic wedge resection of the stomach. Histological examination of the resected sample revealed that the maximum diameter of the tumor was only 6 mm and that dense proliferation of rather uniform spindle tumor cells were observed mainly in the submucosa. Immunohistochemistry showed that they were positive for pan-keratin, CD99 and TLE1. SS18-SSX fusion-specific antibody gave diffuse positive staining to the tumor cells, and analysis using mRNA extracted from paraffin sections revealed that the tumor had SS18-SSX1 fusion gene. Thus, it was diagnosed as gastric synovial sarcoma, monophasic fibrous type.

**Conclusions:**

Primary synovial sarcoma of the stomach is rare and only 47 cases have been reported in the English literature to date. The maximum diameter of the lesion of our case was 6 mm which is the smallest among them.

## Introduction

Synovial sarcomas are a type of high-grade sarcomas that accounts for 5-10% of all soft tissue sarcomas. They develop in all age groups, but preferentially occur in young adults. Although synovial sarcomas arise predominantly from the soft tissues near the large joints of the extremities, they can be rarely found in various organs including the head and neck, lungs, chest wall, heart, kidneys, gastrointestinal tract, reproductive organs, bone and central nervous system [1]. Synovial sarcomas basically contain spindle-shaped tumor cells but show varying degrees of epithelial differentiation. Depending on the histological pattern and the degree of differentiation, they are classified into three types: a monophasic fibrous type consisting of a uniform proliferation of spindle cells without epithelial component; a biphasic type consisting of a mixture of distinct epithelial cells and spindle-shaped cells; and a poorly differentiated type consisting of anaplastic spindle and/or round cells.

The cell origin of synovial sarcomas is still unknown while they have a unique chromosomal translocation t(X;18) (p11; q11). The translocation forms the fusion genes of SS18-SSX1, SS18-SSX2 and SS18-SSX4. The detection of these fusion genes using molecular analyses such as fluorescence in situ hybridization (FISH) and reverse transcription polymerase chain reaction (RT-PCR) is useful for definitive diagnosis. Immunohistochemistry (IHC) using SS18-SSX fusion-specific antibodies could also detect the fusion genes with high sensitivity and specificity [2, 3].

Primary synovial sarcoma of the stomach is rare and only 47 cases have been reported in the English literature to date [4–26]. In this report, we describe a case of small primary gastric synovial sarcoma with ulcer. The gastric tissue obtained by laparoscopic wedge resection revealed that the maximum diameter of the lesion was 6 mm which is the smallest among the primary gastric synovial sarcoma cases reported to date.

## Case report

**Fig. 1:**
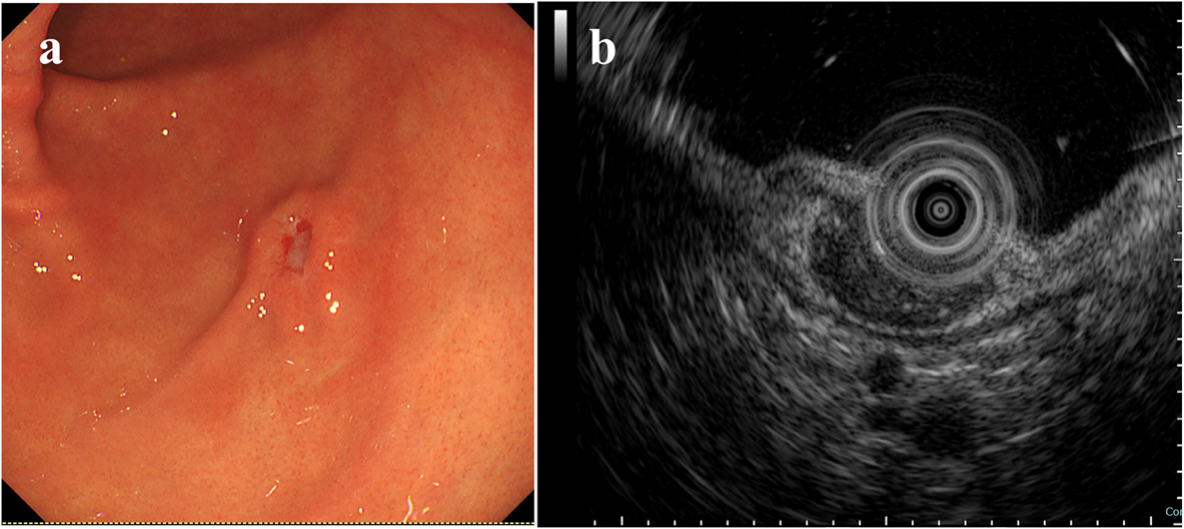
EGD and EUS findings. **A** EGD showed that a tumor with small ulcer located on the greater curvature of the gastric angle. **B** EUS revealed a hypoechoic mass mainly in the third layer (submucosa)

A 61-year-old Japanese woman with a history of appendicitis treated with surgery at the age of 40 was pointed out edematous erosion at the body of the stomach during the regular esophagogastroduodenoscopy (EGD) test at her local doctor. Biopsy was performed with the result that spindle cell proliferation was observed without epithelial atypia. Gastrointestinal stromal tumor (GIST) was suspected and the patient was referred to our hospital for the precise examination. EGD was performed again at our hospital, and a small ulcerative lesion was present at the same area as detected previously (Fig. 1A). Endoscopic ultrasound (EUS) showed a homogenous hypoechoic lesion suspiciously originating from the muscularis mucosae (Fig. 1B). The clinical diagnosis was a subepithelial tumor with ulcer. Biopsy was performed from the ulcerated portion at the apex of the lesion. Hematoxylin and eosin (H&E) staining of the biopsy specimen showed dense proliferation of spindle-shaped tumor cells mixed with smooth muscle fibers of the muscularis mucosae (data not shown). IHC showed that the tumor cells were all negative for KIT, DOG-1, CD34, S-100P, desmin, α-SMA, calponin and caldesmon (data not shown). These immunohistochemical results suggested that the tumor is not GIST, smooth muscle tumors or schwannoma. Ki-67 labeling index of the tumor cells was approximately 10% (data not shown). Although the preoperative histological diagnosis was not clearly determined, some type of malignant tumor was suspected. Thus, the patient underwent laparoscopic wedge resection of the stomach.

**Fig. 2:**
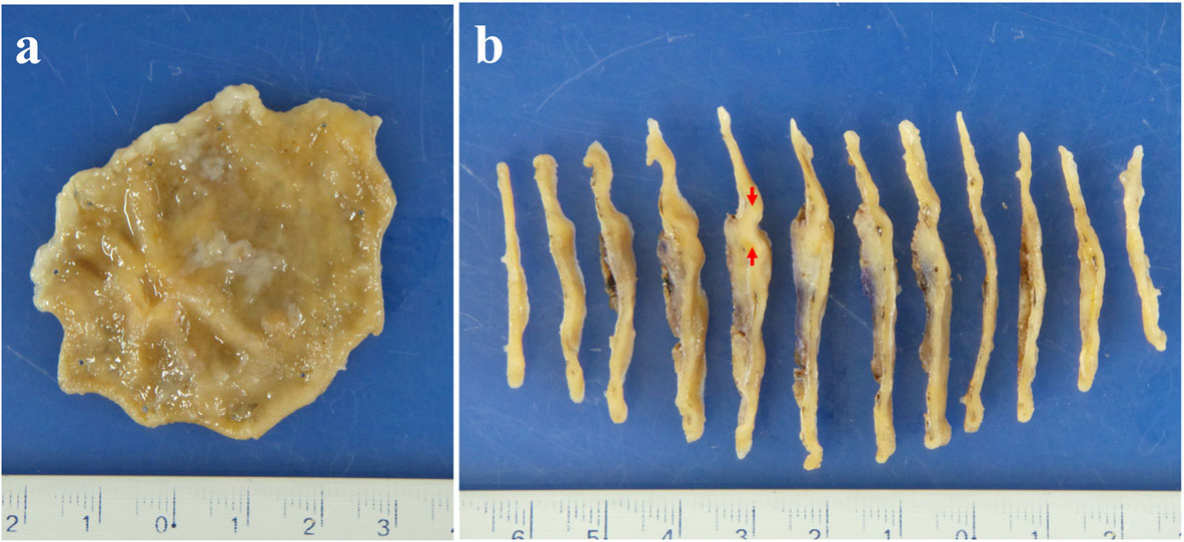
Macroscopic findings of the resected specimen. **A** A mass with ulcer and bridging fold was shown. **B** Cut surface of the specimen showed small white-colored nodule in the submucosa. Red arrows showed the tumor area (maximally 6 mm in size) demonstrated in Fig. 3A

**Fig. 3:**
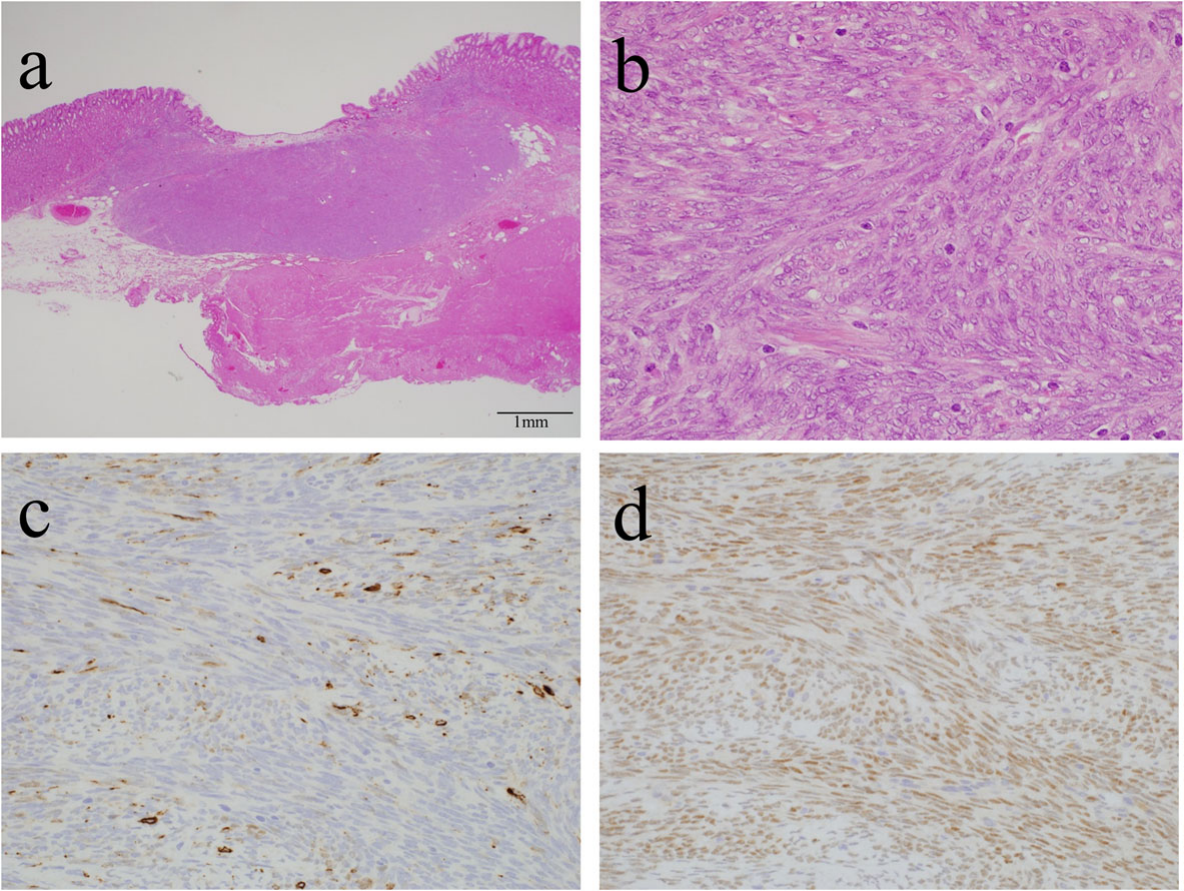
Pathological findings of the tumor. **A** The maximum size of the mass was 6 mm, and it was mainly located in the submucosa (H&E staining, original magnification ×40). **B** It consisted of a dense proliferation of spindle-shaped cells (H&E staining, original magnification ×400). **C** AE1/AE3 was focally positive in tumor cells. **D** SS18-SSX fusion-specific antibody gave diffuse positive staining to tumor cells

**Fig. 4:**
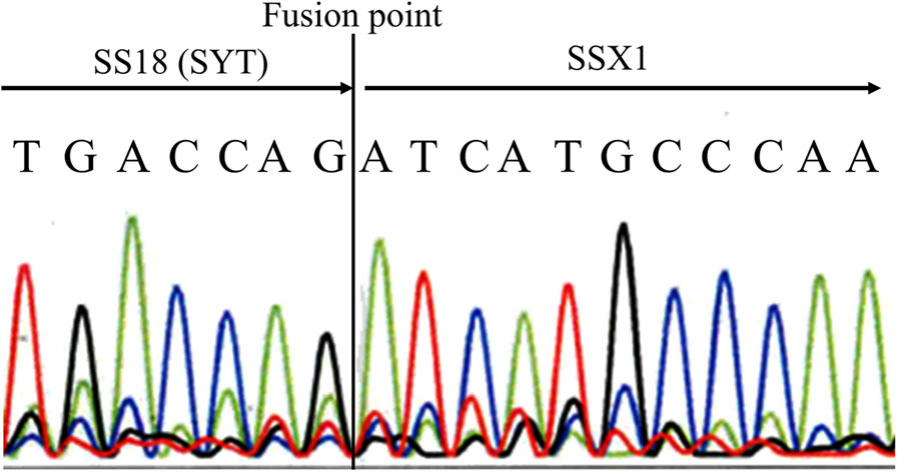
Fusion gene analysis using mRNA extracting from paraffin-embedded tumor section. Fusion of the SS18 gene with the SSX1 gene was detected

On the resected specimen, a slightly elevated lesion with ulcer was observed (Fig. 2A). Cut surface of the lesion showed a small whitish lesion with ulcer (Fig. 2B). H&E staining of the histological section showed dense proliferation of rather uniform spindle tumor cells mainly in the submucosa (Fig. 3A, B). As observed in the biopsy sample, tumor cells were mixed with smooth muscle fibers of the muscularis mucosae. Some of them were also present even in the mucosa. The maximum diameter of the tumor was 6 mm (Fig. 3A). There was no necrosis, vascular invasion and apparent mitotic figures. IHC showed that KIT, DOG-1, CD34, S-100P, desmin and α-SMA were also negative as observed in the biopsy specimen (data not shown). Ki-67 labeling index of the tumor cells in the resected specimen was approximately 5% (data not shown). These results led us to the possible diagnosis of synovial sarcoma, and the tumor cells were shown to be partially positive for AE1/AE3 (Fig. 3C), partially and weakly positive for CD99, and diffusely positive for vimentin and TLE1 by IHC (data not shown). Finally, IHC with SS18-SSX fusion-specific antibody gave diffuse positive staining to the tumor cells (Fig. 3D), and fusion gene analysis using mRNA extracted from paraffin sections revealed that the tumor had SS18 (SYT)-SSX1 fusion gene (Fig. 4). Thus, the tumor was diagnosed as synovial sarcoma, monophasic fibrous type. Since the tumor was so small and the cut ends of the resected sample were considered to be all tumor-free, adjuvant chemotherapy was not planned. The patient had an uneventful postoperative course and was discharged from our hospital. She has no recurrent lesion for 4 months after the surgery.

## Discussion and conclusions

We describe here a case of small primary gastric synovial sarcoma with ulcer occurring in a 61-year-old Japanese woman. Since the description by Billings et al. in 2000 [4], 47 cases of primary synovial sarcoma of the stomach have been reported in the English literature [4–23]. The clinicopathological characteristics of those primary synovial sarcoma of the stomach included age range of 13-72 years, male to female ratio of 23:24, and size range of 8-160 mm (mean 49 mm). Thus, our case had the smallest tumor size among the primary gastric synovial sarcoma cases reported to date. Most of the patients underwent tumor wedge resection or partial gastrectomy, and only two patients underwent total gastrectomy. Metastasis or recurrence was noted in 9 cases, with liver metastasis being the most common in 5 cases, and peritoneal metastasis in 4 cases (2 of which also had liver metastasis). Our patient received wedge resection of the stomach, and has no recurrent or metastatic lesion for 4 months after the surgery.

Histologically, the biphasic type of synovial sarcoma is relatively easy to diagnose due to its characteristic biphasic appearance, while the monophasic type and poorly differentiated type are not so easy to diagnose because many types of spindle cell tumors could be candidates for differential diagnosis. The majority of gastric synovial sarcomas show monophasic type, and the common spindle cell tumors of gastrointestinal tract such as GIST, smooth muscle tumors and schwannoma are included in differential diagnosis. It is difficult to differentiate them by H&E staining alone. Therefore, IHC for general markers for the common spindle cell tumors of gastrointestinal tract such as KIT, DOG1, CD34, S-100P and desmin has to be performed. When these markers are all negative, a possibility of monophasic synovial sarcoma has to be considered. IHC for AE1/AE3, CD99 and TLE1 might be useful for diagnosis, but the definite diagnosis needs detection of SS18-SSX1, SS18-SSX2 or SS18-SSX4 fusion gene by FISH, RT-PCR and/or IHC.

For soft tissue synovial sarcoma, the 5-year survival rate is reported to be 83% in children and adolescents under 19 years of age, 62% in adults, and the 10-year survival rates are 75% and 52%, respectively [1]. The prognosis is good for tumors with a diameter of 5 cm or less, 10 mitoses/10 high power fields (HPFs) or less, no areas of necrosis or poor differentiation. When the tumor size is limited to 1 cm or less, the prognosis is much better [27, 28]. Surgical resection is the mainstay of the treatment, but radiation therapy or chemotherapy might be combined in some cases. Although the efficacy of chemotherapy is still controversial, it has been reported to improve survival in high-risk patients [29]. On the other hand, chemotherapy is not recommended for patients with tumors less than 5 cm in diameter and R0 resections [30]. In the cases of gastric synovial sarcoma, adjuvant chemotherapy was administered in eight patients with tumor diameters of 60 mm or greater [5–7, 13, 20, 22, 24]. Six of eight patients treated with adjuvant chemotherapy had recurrence, and two of these patients died [5, 6].

In recent years, it has become possible to detect very small gastrointestinal tumors with the development of endoscopic devices. Because gastric synovial sarcomas are apt to invade the mucosa, it is often accompanied by ulceration [26]. In our case, the maximum size of the tumor was 6 mm which was the smallest among the gastric synovial sarcomas reported so far, but it still invaded the mucosal layer and showed a hill-like gross image with an apical ulcer. When enlarged, other subepithelial tumors show the same central ulceration, but when small tumors are accompanied by ulceration, the possibility of synovial sarcoma should be kept in mind. The smallest case previously reported was 8 mm in diameter [6], and five cases were in the 10 mm range [6, 15, 22, 25]. All of them underwent wedge resection or partial resection, and none of the described cases were treated with additional therapy, and no recurrence was reported. The smallest one which confirmed recurrence was 20 mm in diameter, and the patient died of the tumor after 2.4 years [6]. All other recurrences or deaths have occurred in relatively large tumors of 50 mm or more (60-160 mm) [4–6, 13, 14, 16].

Because of the small number of reported cases, it is difficult to accurately predict the prognosis of gastric synovial sarcoma, and a treatment strategy has not been established. The accumulation of further cases and long-term follow-up data are desirable.

## Data Availability

The datasets used and analyzed during the current study are available from the corresponding author on reasonable request.

## Abbreviations

IHC: Immunohistochemistry
EGD: Esophagogastroduodenoscopy
GIST: Gastrointestinal stromal tumor
EUS: Endoscopic ultrasound
H&E: Hematoxylin and eosin
FISH: Fluorescence in situ hybridization
RT-PCR: Reverse transcription polymerase chain reaction
